# Real-world deployment and longitudinal evaluation of a human-in-the-loop telemedicine communication support system

**DOI:** 10.3389/fdgth.2026.1864704

**Published:** 2026-09-29

**Authors:** Roland Roller, Ajay Madhavan Ravichandran, Tillmann-Leonhard Breitenstein, Marcel Naik, Bianca Zukunft, Bilgin Osmanodja, Matthias Pfefferkorn, Tim Gielsdorf, Judith Schleder, Sinem Karaterzi, Kristina Boss, Philipp Winnekens, Mario A. Cypko, Aditya Kumar, Ulrich Kunze, Lisa Raithel, Oliver Witzke, Oliver Amft, Lars Pape, Sebastian Möller, Mario Schiffer, Klemens Budde, Wiebke Duettmann

**Affiliations:** 1 Speech and Language Technology Lab, German Research Center for Artificial Intelligence (DFKI), Berlin, Germany; 2 Department of Nephrology and Medical Intensive Care, Charité – Universitätsmedizin Berlin, Berlin, Germany; 3 Department of Infectious Diseases and Nephrology, University Hospital Essen, Essen, Germany; 4 Hahn-Schickard-Gesellschaft für angewandte Forschung e.V., Freiburg, Germany; 5 Intelligent Embedded Systems Lab, University of Freiburg, Freiburg, Germany; 6 Department of Medicine 4 – Nephrology and Hypertension, University Hospital Erlangen, Erlangen, Germany; 7 Quality & Usability Lab, Technische Universität Berlin, Berlin, Germany; 8 BIFOLD – Berlin Institute for the Foundations of Learning and Data, Berlin, Germany; 9 Institute for Artificial Intelligence in Medicine, Charité – Universitätsmedizin Berlin, Berlin, Germany; 10 Vivantes, Auguste Viktoria Krankenhaus, Berlin, Germany; 11 Berlin Institute of Health, Charité – Universitätsmedizin Berlin, Berlin, Germany

**Keywords:** adherence, chat-support, human-in-the-loop, nephrology, telemedicine

## Abstract

**Background:**

Telemedicine platforms are increasingly used to support the long-term monitoring of chronically ill patients. However, continuous streams of patient-reported data and messages may impose substantial workload on telemedicine teams. To address this challenge, we developed a human-in-the-loop communication support system for telemedicine monitoring of kidney transplant recipients.

**Methods:**

The system was integrated into the telemedicine program of the nephrology department at Charité – Universitätsmedizin Berlin. It analyzes telemedicine data in combination with locally stored electronic health record (EHR) data and generates suggested responses and reminders for clinical staff. All suggestions are reviewed by telemedicine personnel and can be accepted, modified, or discarded before being sent to patients. System usage was evaluated based on routine operation data collected since deployment.

**Results:**

The system has been in productive use since May 15, 2024. During the observation period, more than 22,000 message suggestions were generated, of which over 16,000 were sent without modification. Overall, approximately 76% of all generated suggestions resulted in patient communication. Usage increased steadily over time, indicating successful integration into routine telemedicine workflows. Standardized reminders and routine communication tasks showed particularly high utilization.

**Conclusions:**

Our findings demonstrate the potential of automated communication support systems to assist routine telemedicine workflows while maintaining human oversight of patient communication. At the same time, practical challenges related to system integration, data timeliness, and context awareness remain important considerations for successful deployment in clinical environments.

## 1 Introduction

Telemedicine has the potential to become an important component of modern healthcare, particularly for chronically ill patients or those requiring long-term monitoring after complex medical interventions. In nephrology, kidney transplant recipients require continuous monitoring of vital parameters such as blood pressure, weight, and medication adherence.

The telemedicine program SmartNTx^1^ [1] aims to enhance standard post-transplant care through a comprehensive, digital, and data-driven approach. The program integrates mobile health applications, connected measurement devices, and telemedical support to enable continuous monitoring and improved patient self-management. Patients can report vital parameters, well-being, and treatment-related information, which are transmitted to transplant centers via a secure infrastructure.

SmartNTx is implemented as a multicenter intervention involving three German university hospitals (Charité—Universitätsmedizin Berlin, Universitätsklinikum Erlangen, and Universitätsklinikum Essen) and is evaluated in a prospective, randomized controlled trial. The intervention aims to improve clinical outcomes such as medication adherence, blood pressure control, graft function, and reduction of hospitalizations, while also enhancing patient quality of life and enabling more efficient care delivery. In addition to outcome evaluation, the study includes a process evaluation focusing on usability, acceptance, and perceived benefits from both patient and provider perspectives. Within SmartNTx, telemedicine staff monitor incoming patient data streams and communication. Patients regularly submit their vital parameters and self-reported well-being via a mobile application. This data is reviewed by telemedicine teams, who respond to abnormal values, patient inquiries, or missing measurements, and communicate reminders for appointments or medication changes.

Currently, a total of 758 patients are enrolled in the telemedicine monitoring program. At Charité—Universitätsmedizin Berlin, a telemedicine team of three main staff members (telemedicine nurses) is responsible for monitoring patient data streams and managing patient communication. While such systems can improve patient monitoring and facilitate earlier detection of potential risks, they also generate substantial communication workload.

To support this workflow, we developed a human-in-the-loop communication support system that generates message suggestions based on patient data and incoming communication. Importantly, the system does not autonomously communicate with patients and does not function as a fully autonomous chatbot. Instead, all generated messages must be reviewed and approved by telemedicine staff before being sent. The functionality was integrated into the working environment of the telemedicine team at Charité—Universitätsmedizin Berlin and introduced into routine operation on May 15, 2024. The development and evaluation of the system were conducted within the SmartNTx+ nested research study, which accompanies the multicenter SmartNTx telemedicine trial [1]. The present work reports a retrospective analysis of operational system logs generated during routine use of the communication support system. It describes the system architecture, reports real-world usage statistics, and summarizes lessons learned from integrating a human-in-the-loop communication support system into clinical telemedicine workflows.

## 2 System overview

### 2.1 Overall architecture

The telemedicine platform used in SmartNTx enables kidney transplant recipients to regularly report health-related data through a mobile application [2]. Depending on individual treatment plans, patients submit measurements such as blood pressure, weight, and self-reported well-being at predefined intervals (e.g., daily, weekly, or bi-weekly). In addition, the platform provides a chat-based communication channel that allows patients to contact the telemedicine team and receive feedback, reminders, or clinical instructions. An overview of the system architecture and interaction between patients, telemedicine staff, and the communication support system is shown in Figure 1.

**Figure 1:**
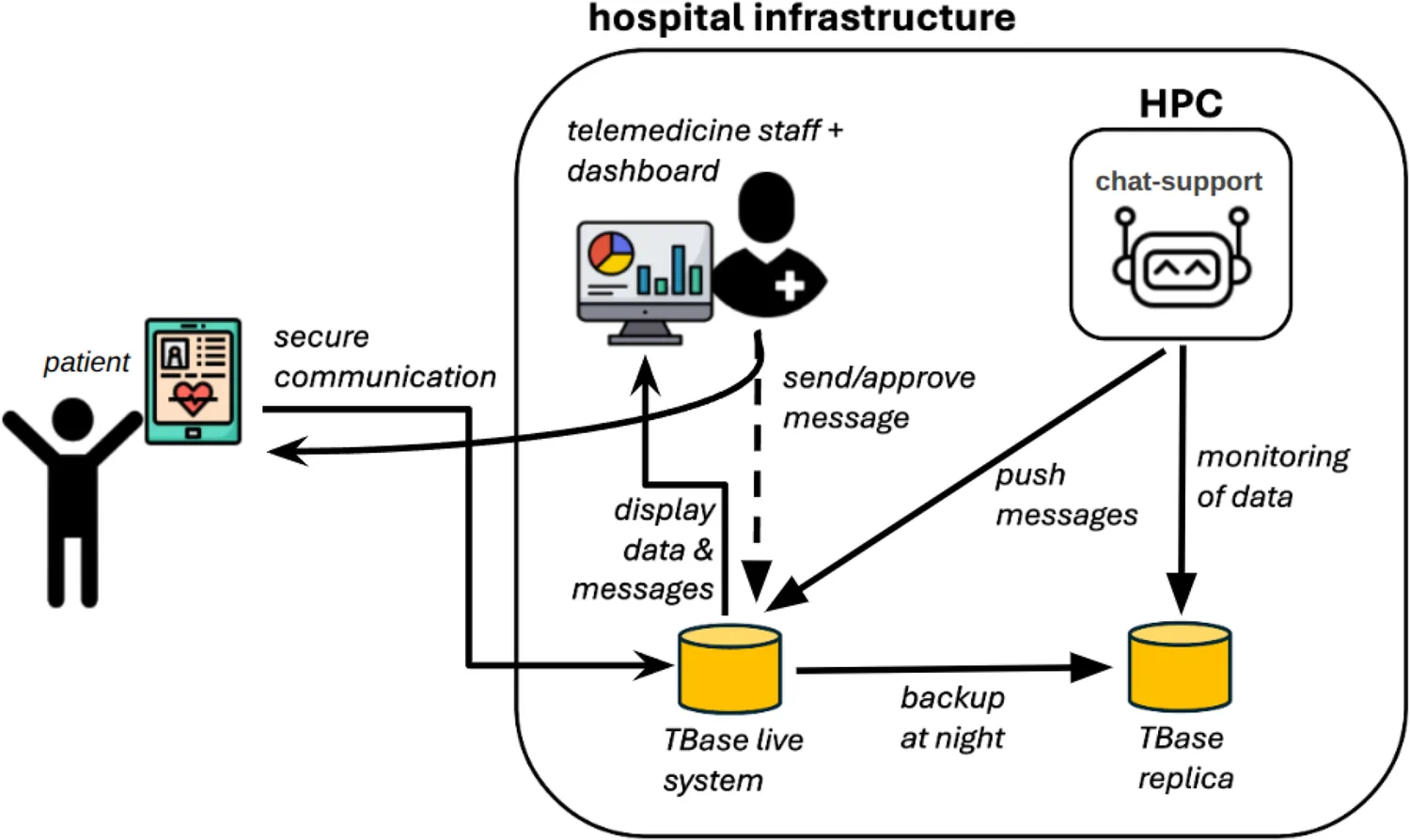
Overview of architecture and the interaction of patient, telemedicine staff and communication support system.

All patient-reported data and messages are transmitted via a secure FHIR-based interface into the clinical documentation system TBase [3], which serves as the central working environment for telemedicine staff. Within this system, incoming data streams and patient messages are continuously reviewed and managed by the telemedicine team.

The communication support system was implemented as an additional service running on the high-performance computing (HPC) infrastructure of Charité—Universitätsmedizin Berlin. Due to data protection and system access restrictions, the service does not directly interact with the live clinical system. Instead, it operates on a replicated version of the TBase database, which is updated once per day (4 AM). After that, an automated processing pipeline analyzes the replicated data and identifies events that may require communication with patients. Based on this analysis, the system generates message suggestions, which are subsequently transferred to the live system via a dedicated interface. The generated suggestions are integrated directly into the telemedicine user interface, where they are presented alongside patient data. Telemedicine staff can review each suggestion and decide whether to send it unchanged, modify the content, or discard it. Suggestions that are not used are automatically removed after five days. Note, that our architecture, with a daily database update at 4 AM, simplifies integration and data protection but may result in suggestions being generated from data that are no longer fully up-to-date at the time of manual review.

This design ensures that the system supports clinical workflows without replacing human decision-making, while allowing efficient handling of routine communication tasks.

### 2.2 System deployment and evaluation design

The communication support system was introduced into routine operation at Charité—Universitätsmedizin Berlin on May 15, 2024 and continuously used as part of the SmartNTx+ nested study. The present work reports a retrospective observational analysis of operational system logs collected during routine use between May 15, 2024 and March 16, 2026.

All generated message suggestions during this period were included in the analysis. The logs contain timestamps, message categories, and the action performed by telemedicine staff after reviewing each suggestion. For each generated suggestion, telemedicine staff could either (1) send the message without modification, (2) modify the suggested text before sending, or (3) discard the suggestion. Message adoption rate was defined as the proportion of generated suggestions that were ultimately transmitted to patients, either unchanged or after modification. Analyses were performed retrospectively on pseudonymized operational log data and are descriptive in nature.

## 3 Message generation approach

While recent advances in large language models (LLMs) enable highly flexible text generation, their direct use in clinical communication raises concerns regarding reliability, transparency, and controllability. Therefore, the communication support system follows a controlled semi-automated strategy based on predefined message templates rather than unrestricted text generation.

The templates were initially created with the assistance of LLMs and subsequently reviewed and iteratively refined together with clinicians and telemedicine staff during pre-deployment testing and early use. This process ensured that the resulting messages were clinically appropriate, understandable, and aligned with routine communication practices.

The system currently supports three categories of messages:

- reminders for missing measurements,
- appointment reminders, and
- notifications concerning abnormal or potentially critical values.

Message generation is triggered by predefined rules derived from patient measurements and administrative events. Thresholds used for the detection of abnormal vital signs are summarized in Table 1 [2]. Blood pressure, heart rate, temperature, and self-reported well-being are categorized into normal, abnormal, and critical ranges. Weight changes are evaluated over multiple time windows (1, 3, and 8 days) to capture both short-term fluctuations and more gradual trends. Well-being is assessed using a Likert-type scale ranging from 1 (very good) to 5 (very poor). Only critical events trigger messages.

**Table 1:** Classification thresholds for vital signs and patient-reported outcomes.

Parameter	Critical	Abnormal	Normal	Abnormal	Critical
Systolic BP	<90 mmHg	90–99 mmHg	100–129 mmHg	130–180 mmHg	>180 mmHg
Diastolic BP	<50 mmHg	50–59 mmHg	60–89 mmHg	90–100 mmHg	>100 mmHg
Heart Rate	<50 bpm	50–59 bpm	60–89 bpm	90–120 bpm	>120 bpm
Temperature	$<33.5^{\circ }C$	33.5–36.2°C	36.3–37.4°C	37.5–38.0°C	$>38.0^{\circ }C$
Weight Change (1 day)	>−1.5 kg	−1.5 to −0.5 kg	±0.5 kg	0.5–1.5 kg	>1.5 kg
Weight Change (3 days)	>−2.5 kg	−2.5 to −1.0 kg	±1.0 kg	1.0–2.5 kg	>2.5 kg
Weight Change (8 days)	>−3.0 kg	−3.0 to −1.5 kg	±1.5 kg	1.5–3.0 kg	>3.0 kg
Well-being Score			1–2 points	3–4 points	5 points

For each message category, several template variants are available. In the German version, 10 differnt templates are available for appointment reminders and reminders for missing measurements, and three templates for notifications regarding abnormal values. Corresponding English templates are available for bilingual communication. Additional contextual variants are selected depending on the affected parameter, the severity of an abnormality, and administrative circumstances such as the beginning of a new quarter.

To avoid repetitive communication patterns, one template variation is randomly selected each day and subsequently applied to all patients receiving messages of the same category on that day. Patient-specific information, such as appointment dates or abnormal measurement values, is inserted automatically. Representative examples of generated message suggestions are shown in Table 2. For messages regarding abnormal values, the generated content additionally depends on the affected parameter and the severity of the deviation. All templates were reviewed and approved by clinical staff before deployment and may be updated over time based on practical experience and user feedback. Thus, the system combines the advantages of LLM-assisted content creation with the transparency, predictability, and safety of template-based communication.

**Table 2:** English text examples generated as suggestions for telemedicine staff.

Category	Example
Appointment reminders	Hello,
	we would like to take this opportunity to inform you about your next appointment
	at the kidney transplant clinic on October 16, 2025.
	Since a new quarter has begun, please remember to bring referral and your
	insurance card.
	We look forward to seeing you, your outpatient care teams.
Missing measurements	Hello,
	I hope that you are doing well.
	I would like to kindly remind you to please enter your vital signs in the Easy
	App again and send them to us.
	If you have any questions, you can reach us Monday to Friday from 08:00 to
	15:00 on 030 450 514213.
	Best regards from your telemedicine team
Abnormal values	Good morning,
	We have noticed that your temperature is too high. Could you please confirm
	whether this reading is correct or if it is a data entry error?
	Kind regards, your telemedicine team

## 4 Results

The communication support system has been in productive use since May 15, 2024. During the observation period (until March 16, 2026), a total of 22,157 message suggestions were generated. Of these, 16,006 messages were sent without modification and 815 messages were modified before sending, resulting in an overall adoption rate of 75.9%. For the purpose of this analysis, both messages sent without modification and messages modified before sending were considered accepted, whereas discarded or expired suggestions that were not sent within five days were classified as not adopted. On average, the system generated approximately 1,021 message suggestions per month, of which around 772 were ultimately used by telemedicine staff.

### 4.1 Overall system usage

Figure 2 shows the monthly number of generated and used message suggestions. After an initial phase with several hundred generated messages per month, usage increased steadily and stabilized at approximately 1,100–1,200 generated messages per month. The number of messages ultimately sent by telemedicine staff followed a similar trend, indicating increasing integration of the system into routine workflows. Temporary decreases in some months were attributable to periods of system unavailability caused by maintenance, infrastructure issues, and administrative delays in restoring access. For example, in June 2025, communication support was unavailable for several days because server access had to be renewed.

**Figure 2:**
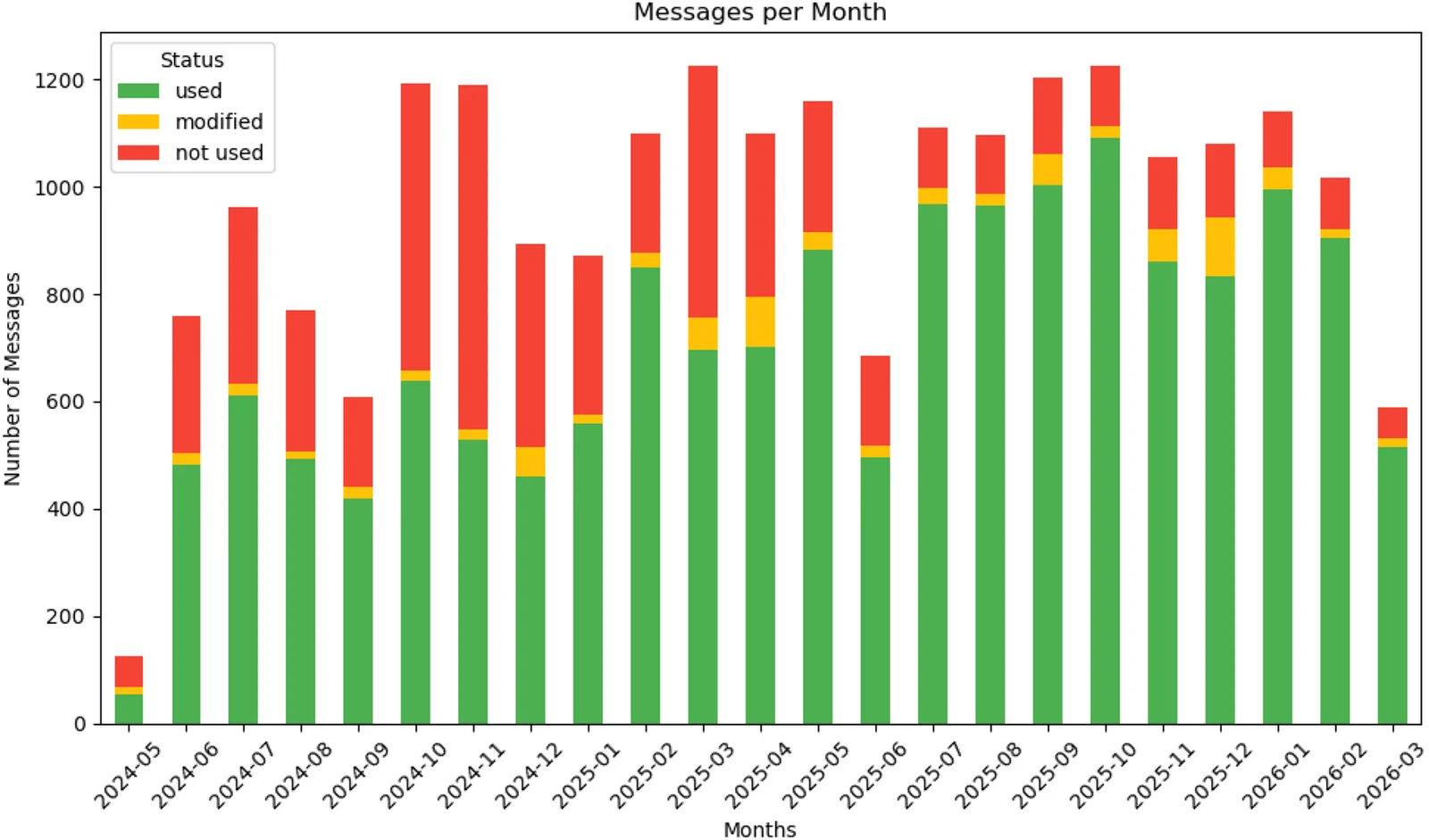
Overview of generated and used message suggestions per month. The communication support system was introduced on May 15, 2024.

The overall communication activity is illustrated in Figure 3. Following the introduction of the communication support system, the total number of messages sent by telemedicine staff increased substantially, from fewer than 200 messages per month to more than 1,000 messages per month. While causality cannot be inferred from this observational analysis, the findings suggest an association between the introduction of the system and an increase in communication frequency and structure.

**Figure 3:**
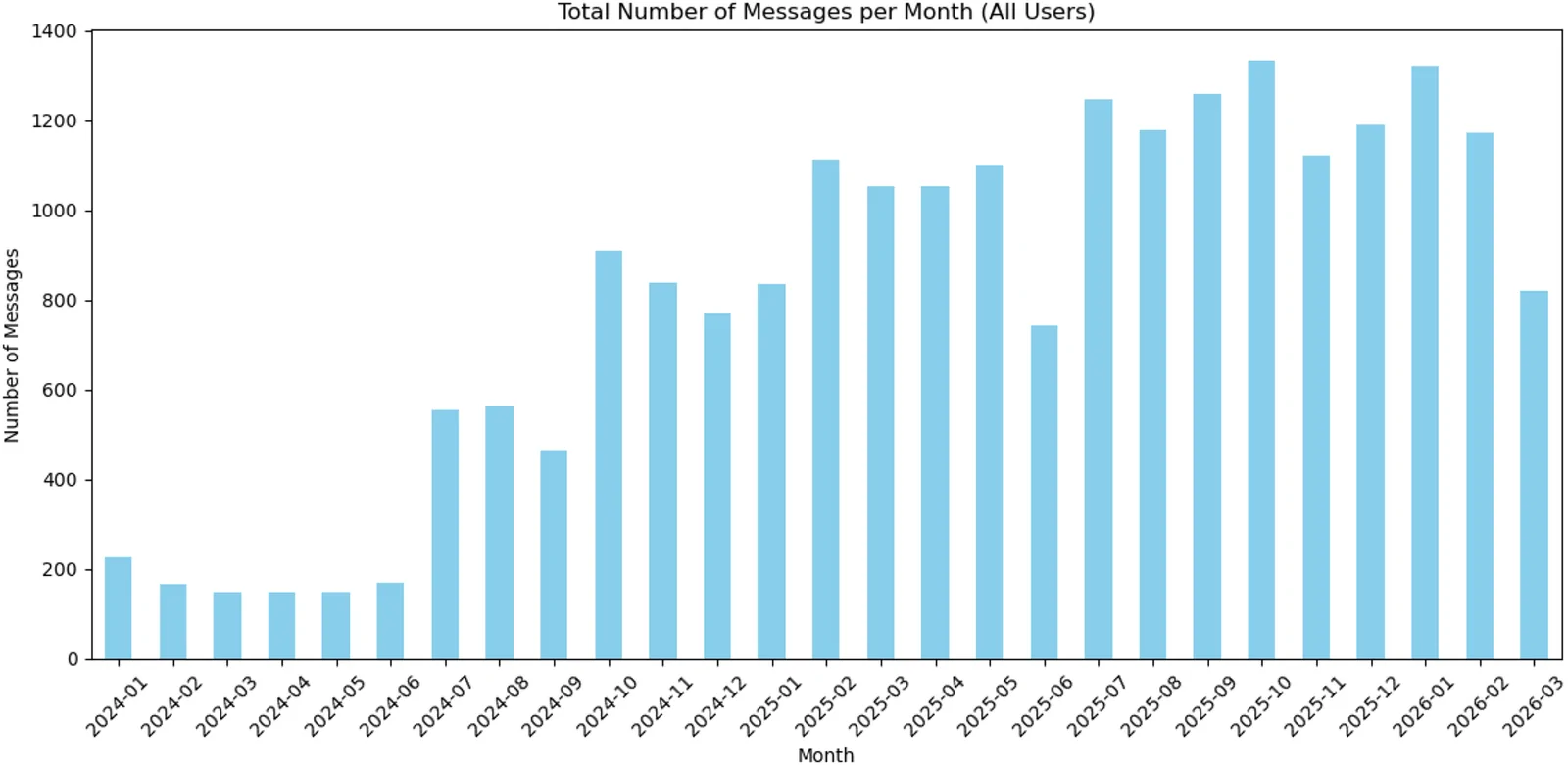
Overall number of messages sent by telemedicine staff per month.

Figure 4 presents the proportion of system-generated messages among all messages sent within Charité during the first year after deployment. Starting from approximately 15% shortly after deployment, the proportion increased rapidly and stabilized at around 70%, indicating growing reliance on the system for routine communication tasks.

**Figure 4:**
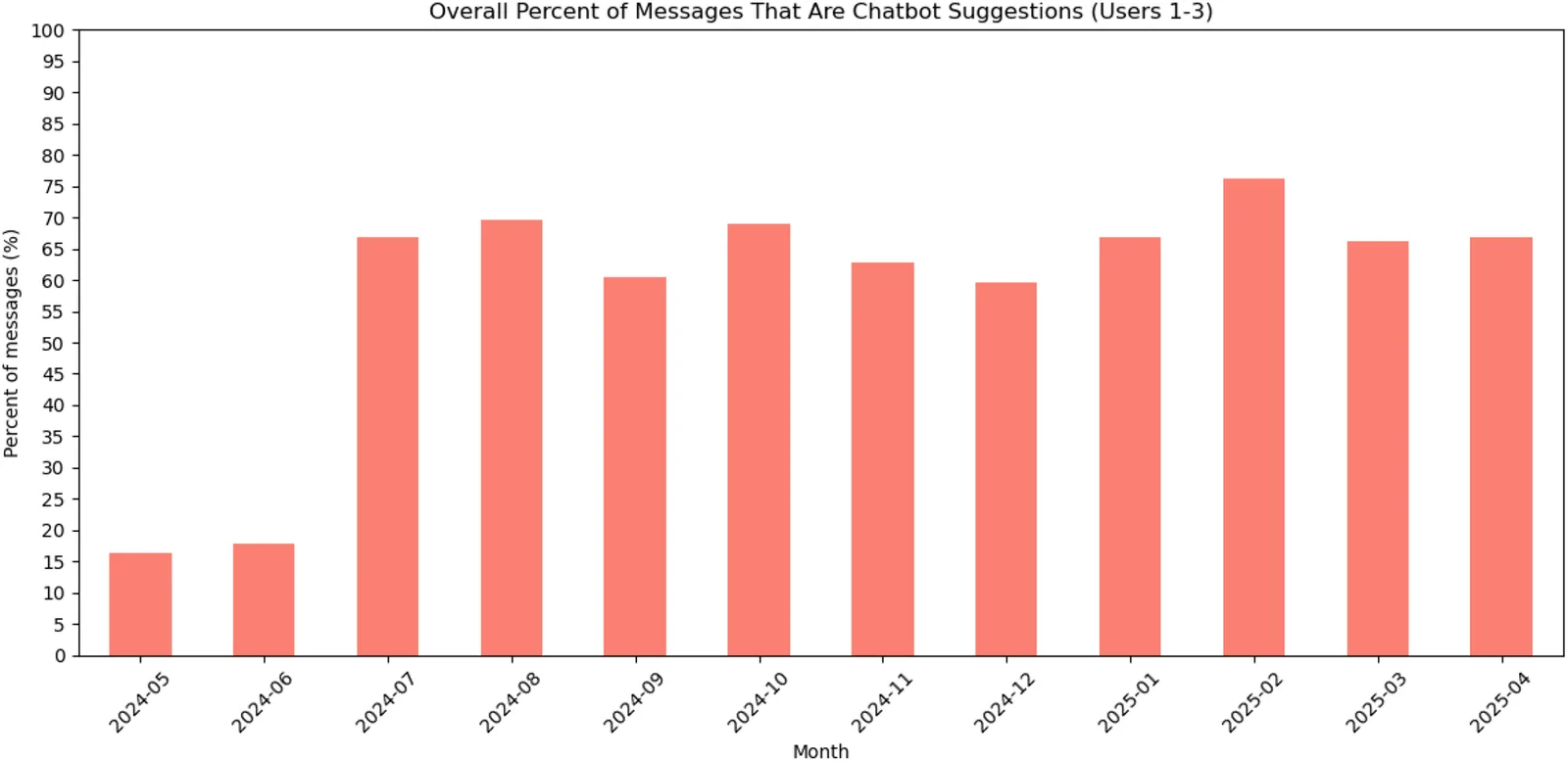
Percentage of communication support system-generated messages among all messages sent by the main telemedicine staff during the first year after deployment.

Figure 5 shows usage patterns for the three telemedicine nurses during the first year. Although initial adoption differed between users, all staff members increased their use of the system over time. Individual differences persisted, with long-term usage rates ranging between approximately 60% and 80%, suggesting that personal preferences and workflow integration influenced the degree of system adoption.

**Figure 5:**
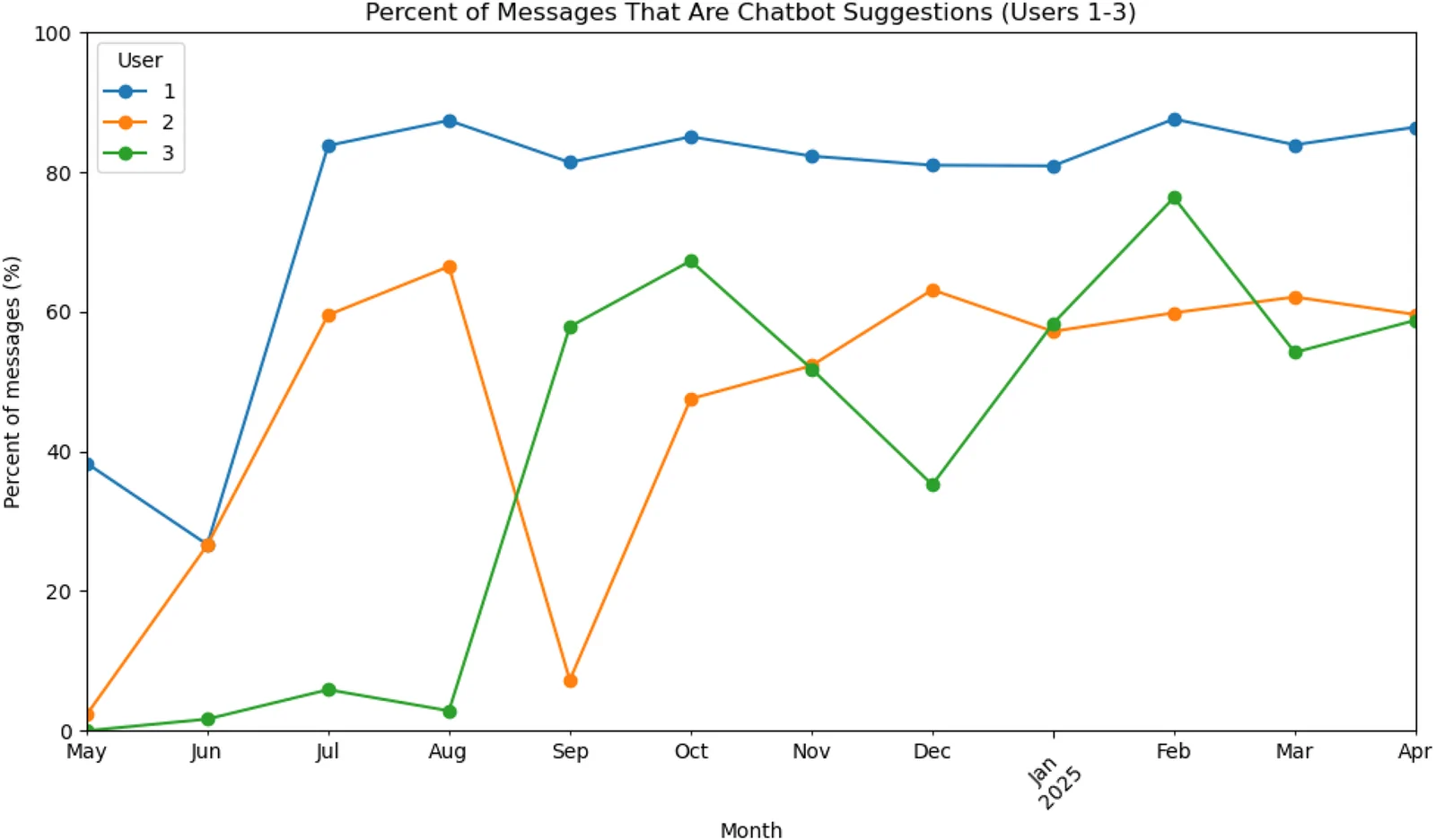
Individual usage patterns of the three telemedicine nurses during the first year after deployment.

### 4.2 Message type-specific adoption

Table 3 provides a breakdown of generated message suggestions by category. Missing measurement reminders represented the largest group (14,240 messages, 64.3%), followed by appointment reminders (4,949 messages, 22.3%) and messages related to abnormal values (2,968 messages, 13.4%).

**Table 3:** Detailed overview about usage of telemedicine of different message types.

Category	Frequency	Adoption rate	Sent unchanged	Modified	Not used
Appointment reminders	4,949	97.8%	90.4%	7.5%	2.2%
Missing measurements	14,240	79.9%	77.6%	2.3%	20.1%
Abnormal values	2,968	20.4%	16.3%	4.1%	79.6%
All messages	22,157	75.9%	72.2%	3.7%	24.1%

Adoption rates differed substantially between message types. Appointment reminders showed the highest overall adoption rate (97.8%), with only a very small proportion of suggestions being discarded. Missing measurement reminders achieved an adoption rate of 79.9%. In contrast, messages concerning abnormal values exhibited a considerably lower adoption rate of 20.4%, with the majority of suggestions not being used. Across all categories, 75.9% of generated suggestions ultimately resulted in a message being sent, while 72.2% were sent without modification and 3.7% were modified before transmission.

### 4.3 Qualitative analysis of modified messages

To better understand the nature of message adaptations, an exploratory manual review of a sample of modified suggestions (20 examples per message category) was performed.

For reminders concerning missing measurements and appointments, modifications were predominantly minor and mainly consisted of wording adjustments, additional personalization, or inclusion of contextual information such as holidays, birthdays, or appointment details. In some cases, appointment reminders were combined with additional requests to regularly submit measurements. Overall, these changes were largely stylistic and aimed at improving the tone and clarity of the communication.

In contrast, modifications of messages concerning abnormal values were substantially more extensive. Telemedicine staff frequently added patient-specific context, clarified which measurements and time periods were affected, referred to previous contacts or telephone calls, requested additional findings from external physicians, and, in some cases, increased the urgency of recommendations. Several modifications reflected knowledge about the patient’s current situation and ongoing care trajectory that was not available to the communication support system.

Taken together, these findings indicate that the degree of required human intervention differs substantially across communication tasks. Routine reminders are frequently suitable for direct use with only minor stylistic adjustments, whereas communication regarding abnormal or potentially critical values often requires additional contextual information and clinical interpretation. This pattern is consistent with the lower adoption rates observed for messages concerning abnormal values.

### 4.4 Temporal evolution of adoption rates

To investigate whether usage patterns changed over time, monthly adoption rates were analyzed separately for each message category. Figure 6 illustrates the temporal evolution of adoption rates.

**Figure 6:**
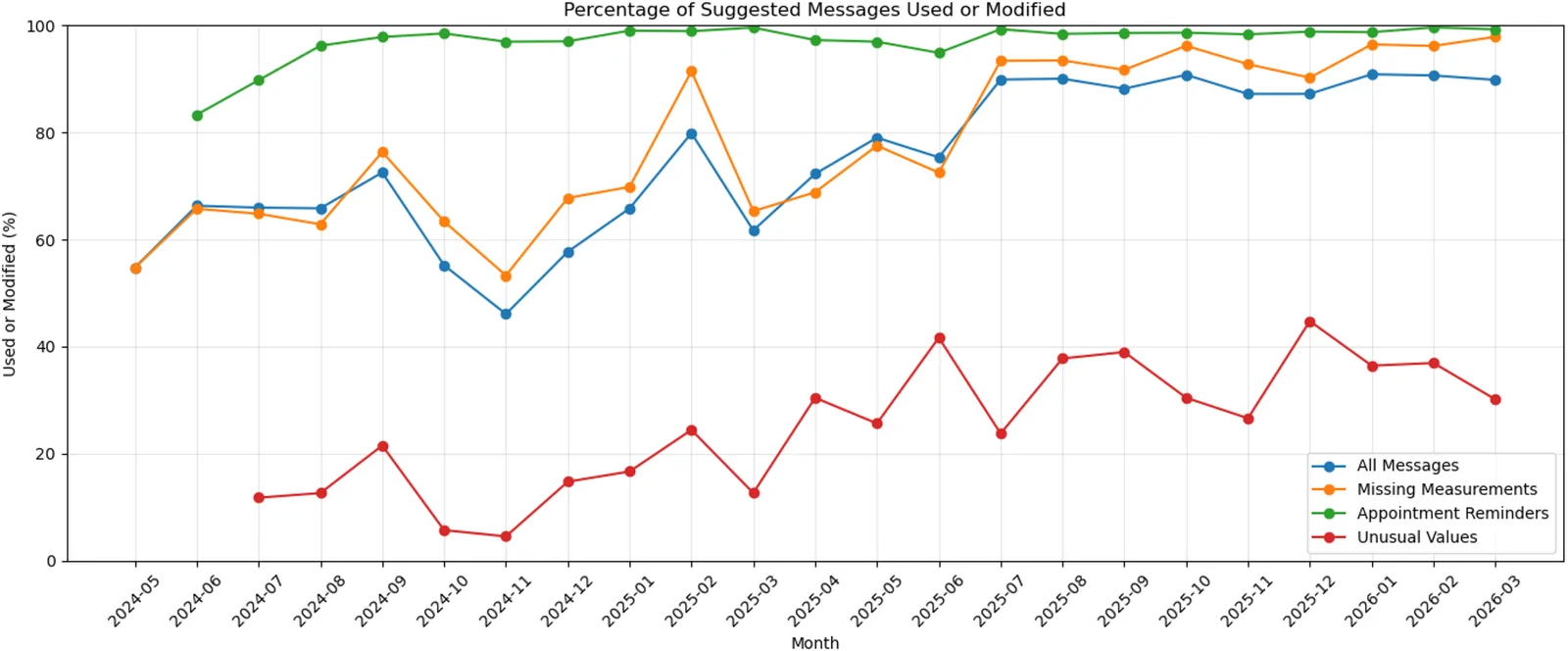
Message type adoption rate over time.

Overall adoption increased from approximately 55%–66% during the first months after deployment to around 90% in the final observation period. A similar trend was observed for missing measurement reminders, whose adoption rate increased from approximately 55%–65% to more than 95%. Appointment reminders exhibited consistently high adoption rates throughout the study period, remaining close to 100% across almost all months. Messages concerning abnormal values showed substantially lower and more variable adoption rates, although a moderate increase over time was also observed.

These findings suggest considerable differences in the suitability of different communication tasks for suggestion-based support. Routine and administrative communication tasks appear particularly amenable to semi-automated support, whereas messages concerning abnormal values continue to require substantial human oversight and contextualization.

Here’s a tightened version of the Discussion section. I’ve preserved all key findings and arguments while cutting redundant phrasing and merging closely related points:

## 5 Discussion

The results demonstrate that the system can support telemedicine workflows, particularly for standardized communication tasks. The high proportion of messages sent without modification suggests that many routine interactions, such as reminders for missing measurements or upcoming appointments, can be reliably supported through suggestion-based systems. Moreover, the observed increase in overall communication volume and the proportion of system-supported messages over time indicates a gradual integration into clinical practice. This pattern suggests increasing familiarity and trust in the system, consistent with growing adoption by telemedicine staff.

Qualitative feedback confirmed that appointment reminders and reminders for missing measurements were perceived as especially useful, with the latter providing notable time savings compared to manual e-mail communication. Feedback further highlighted clear differences across task types. Routine reminders typically required only minor wording adjustments, whereas messages concerning abnormal or potentially critical values more often required substantial adaptation incorporating patient-specific context, such as prior contacts, chronic abnormalities, or information from external physicians.

Several reasons were identified for discarding suggestions, including already updated patient data, prior contact, or lack of clinical relevance. Known patient-specific circumstances (e.g., chronic abnormalities or temporary monitoring interruptions) also frequently rendered suggestions unnecessary, contributing to the lower adoption rates observed for abnormal-value messages.

A key limitation is the temporal delay introduced by daily database replication, which means suggestions may be based on outdated information. This particularly affects time-sensitive situations such as fever or impaired well-being, where telephone contact often precedes message generation. Both log analysis and staff feedback indicate that this leads to a non-negligible number of obsolete suggestions. Moreover, the results underscore the importance of human oversight, which staff considered indispensable before any transmission.

Overall, system performance depends on timely data integration, improved context awareness, and tighter integration with existing clinical workflows. Future work includes individualized thresholds, more real-time processing, and better use of longitudinal clinical notes. Given that all suggestions required staff review before transmission, the system should be understood as a workflow- and decision-support tool rather than an autonomous agent. Hybrid approaches combining automated suggestions with human oversight appear well suited to balancing efficiency and safety in clinical communication.

## 6 Lessons learned and deployment barriers

The deployment of AI-supported tools in clinical environments involves challenges that extend beyond technical implementation. Based on our experience, several practical barriers were identified.

**First**, integration across multiple hospitals is constrained by heterogeneous IT infrastructures and administrative procedures. Differences in security policies, authentication systems, and access management require separate approvals and contractual agreements, which can substantially delay deployment and limit scalability.

**Second**, strict data protection requirements prevent direct access to live clinical systems. In our setup, the communication support system operates on a replicated database updated once per day. While this architecture ensures data security and system stability, it introduces latency between patient data changes and message generation. As reflected in the Results, this delay particularly affects time-sensitive and abnormal-value messages, which were frequently discarded or already addressed by telemedicine staff before review. More frequent data synchronization could improve responsiveness in such cases.

**Third**, integration into clinical workflows requires continuous adaptation to user needs. While the system was designed as a decision-support tool, it introduces an additional interface that must be actively managed. Iterative co-development with telemedicine staff was essential to ensure usability. Qualitative feedback and usage patterns indicate that routine tasks (e.g., appointment and measurement reminders) are more consistently adopted than messages related to abnormal clinical values, which require substantial contextual interpretation.

**Finally**, dependencies on external systems and development cycles can limit scalability. Although the system was designed for deployment across multiple clinical sites, differences in local infrastructure and delayed software integration have so far restricted broader rollout.

These findings highlight that successful deployment of AI-supported clinical communication systems depends not only on technical performance, but also on organizational, infrastructural, and regulatory factors. In particular, our results suggest that system value is highest for structured, low-ambiguity communication tasks, whereas clinically complex situations require richer contextual integration and stronger human oversight.

## 7 Related work

Telemedicine and digital health technologies have been widely studied for their potential to improve chronic disease management [4], patient engagement [5], and continuity of care [6]. Early research identified both opportunities and barriers in remote monitoring and communication, including clinician workload, data workflows, and system integration challenges [7]. More recent work highlights the rapid expansion of telemedicine and its implications for healthcare delivery and clinician workload [8, 9].

### 7.1 Conversational agents and chatbots in healthcare

Previous work has examined the use of conversational agents (chatbots) in healthcare contexts. Systematic reviews document applications in behavior change support, health education, symptom triage, and patient monitoring [10, 11] and present their potential and risks and challenges [12]. Evidence suggests that conversational agents can be feasible and acceptable, but rigorous clinical evaluation remains limited. The present work differs from these studies in that it focuses on deployment within a real-world telemedicine workflow and the integration of communication support into routine clinical operations rather than standalone patient-facing chatbot interventions.

While some randomized controlled trials (RCTs) exist, they are relatively few and focus primarily on mental health or behavioral interventions rather than clinical workflow support. For example, Fitzpatrick et al. [13] demonstrated the effectiveness of a mental health chatbot in reducing depressive symptoms, simlarly as [14]. However, patient safety, long-term outcomes, and especially integration into routine clinical workflows remain underexplored in these systems. Most studies are descriptive or proof-of-concept, focusing on development or usability rather than deployment in routine care. Examples include chatbots for telemedicine support, health information retrieval, and domain-specific diagnostic assistance [15]. The rise of LLMs have further expanded the potential of conversational systems in healthcare, but their safe and effective integration into clinical practice remains an open challenge [16, 17].

### 7.2 Telemedicine chatbots and clinical evaluation

Several studies describe chatbots integrated into telemedicine workflows, including systems for automated reminders, triage, and patient interaction [18, 19]. However, these systems are typically evaluated in pilot settings without rigorous clinical validation. More recent work explores LLM-based telemedicine assistants and knowledge-based systems, but these are often limited to prototype implementations or offline evaluations [20]. Robust evidence from real-world clinical deployment remains scarce. In contrast to prior work, our study focuses on a long-term deployed system embedded in routine clinical practice, with analysis of usage and adoption over an extended operational period rather than short-term pilot evaluation.

### 7.3 Specific domains: nephrology and monitoring

In nephrology, telemedicine has been applied to monitor kidney transplant recipients and support adherence to treatment plans. An overview is given in Mata-Lima et al. [21], Sugawara et al. [22], Ellis et al. [23]. Reported benefits include improved accessibility, reduced patient burden, and enhanced monitoring capabilities. However, existing systems primarily focus on data collection, visualization, and alerting rather than supporting clinician-patient communication through integrated, workflow-based communication assistance and longitudinal deployment in routine care.

### 7.4 Automated monitoring and decision support

Automated monitoring and decision support systems aim to assist clinicians in handling large volumes of patient-generated data. Approaches range from rule-based alert systems to AI-driven triage tools and clinical decision support systems. Prior work has highlighted the increasing burden of digital communication and inbox management in clinical practice, emphasizing the need for tools that support efficient handling of patient messages [24]. While AI-based approaches show promise, many systems remain technically focused and are not fully integrated into clinical workflows or evaluated in real-world care settings. The present study addresses this gap by reporting a deployed human-in-the-loop communication support system integrated into clinical workflow, evaluated through longitudinal usage and adoption data in routine care.

### 7.5 Gap and positioning of this work

Overall, although a large range of research exists on telemedicine, conversational agents, and AI-based decision support, relatively few studies report on systems that are deployed in routine clinical workflows and evaluated over extended periods of real-world use. Existing work predominantly focuses on patient-facing applications, short-term pilots, or controlled experimental settings. In contrast, the present study investigates a human-in-the-loop communication support system embedded in routine telemedicine care for kidney transplant patients. The system has been continuously used in clinical practice over an extended period, enabling analysis of real-world usage patterns, adoption behavior, and workflow integration. This work contributes (i) evidence from long-term real-world deployment, (ii) analysis of clinician adoption and interaction patterns, and (iii) insights into practical challenges of integrating communication support systems into clinical telemedicine workflows.

## 8 Conclusion

In this work, we presented a chat-support system designed to assist telemedicine staff in monitoring kidney transplant patients within the SmartNTx program. The system generates message suggestions based on patient data and supports routine communication tasks while maintaining full human control over patient interaction. Results from more than one year of productive use demonstrate substantial system utilization and increasing integration into clinical workflows over time. In particular, the system appears well suited for standardized communication tasks such as reminders and routine notifications. At the same time, our findings highlight important challenges for the deployment of AI-supported systems in clinical environments. Limitations related to data timeliness, context awareness, and system integration can affect the relevance of generated suggestions and increase the need for manual review. Addressing these challenges will be critical for further improving the usability and impact of such systems.

Future work will focus on enhancing system responsiveness, improving context-sensitive message generation, and extending integration with clinical and telemedicine infrastructures. In addition, further evaluation is needed to quantify the impact on workload, communication quality, and patient outcomes in telemedicine care. Future evaluation approaches may include workload assessments (e.g., time-to-response or message handling time), structured user studies with telemedicine staff, and patient-reported experience measures to better capture communication quality and perceived care impact.

## Data Availability

The data analyzed in this study is subject to the following licenses/restrictions: the patient data cannot be shared without additional approval. Requests to access these datasets should be directed to roland.roller@dfki.de.

## Footnotes

^1^https://www.smartntx.de/.
